# Machine learning integrations develop an antigen-presenting-cells and T-Cells-Infiltration derived LncRNA signature for improving clinical outcomes in hepatocellular carcinoma

**DOI:** 10.1186/s12885-023-10766-w

**Published:** 2023-03-28

**Authors:** Xiaodong Wang, Ji Chen, Lifan Lin, Yifei Li, Qiqi Tao, Zhichao Lang, Jianjian Zheng, Zhengping Yu

**Affiliations:** 1 Zhejiang Provincial Key Laboratory for Accurate Diagnosis and Treatment of Chronic Liver Diseases, The First Affiliated Hospital of Wenzhou Medical University, Wenzhou, 325000 China; 2 Key Laboratory of Clinical Laboratory Diagnosis and Translational Research of Zhejiang Province, The First Affiliated Hospital of Wenzhou Medical University, No.2 Fuxue Lane, Wenzhou, Zhejiang P.R. China; 3 Department of Hepatobiliary Surgery, The First Affiliated Hospital of Wenzhou Medical University, No.2 Fuxue Lane, Wenzhou, Zhejiang P.R. China

**Keywords:** Machine learning, LncRNA signature, Hepatocellular carcinoma, T cell infiltration, Prognosis

## Abstract

As a highly heterogeneous cancer, the prognostic stratification and personalized management of hepatocellular carcinoma (HCC) are still challenging. Recently, Antigen-presenting-cells (APCs) and T-cells-infiltration (TCI) have been reported to be implicated in modifying immunology in HCC. Nevertheless, the clinical value of APCs and TCI-related long non-coding RNAs (LncRNAs) in the clinical outcomes and precision treatment of HCC is still obscure. In this study, a total of 805 HCC patients were enrolled from three public datasets and an external clinical cohort. 5 machine learning (ML) algorithms were transformed into 15 kinds of ML integrations, which was used to construct the preliminary APC-TCI related LncRNA signature (ATLS). According to the criterion with the largest average C-index in the validation sets, the optimal ML integration was selected to construct the optimal ATLS. By incorporating several vital clinical characteristics and molecular features for comparison, ATLS was demonstrated to have a relatively more significantly superior predictive capacity. Additionally, it was found that the patients with high ATLS score had dismal prognosis, relatively high frequency of tumor mutation, remarkable immune activation, high expression levels of T cell proliferation regulators and anti-PD-L1 response as well as extraordinary sensitivity to Oxaliplatin/Fluorouracil/Lenvatinib. In conclusion, ATLS may serve as a robust and powerful biomarker for improving the clinical outcomes and precision treatment of HCC.

**Supplementary Information:**

The online version contains supplementary material available at 10.1186/s12885-023-10766-w.

## Introduction

Hepatocellular carcinoma (HCC), as the major histological subtype of liver cancer, is one of the most common cause of cancer-related mortality worldwide [1]. With high rates of both recurrence and metastasis, the prognosis of advanced HCC patients was poor [2, 3]. Currently, transplantation remains the most effective treatment for HCC [4, 5]. However, the prognosis of HCC patients after transplantation still remains poor because of the differences in tumor burden and liver function [6, 7]. Previously, TNM stage is considered as the universal criteria for cancer staging [8, 9]. Barcelona Clinic Liver cancer (BCLC) system, as the specific liver cancer clinical staging system, also plays a key role in evaluating the prognosis of HCC [10]. The establishment of BCLC staging system has narrowed the difference regarding the management of patients with HCC and is currently the most widely used staging system [11]. In China, the China liver cancer (CNLC) staging system is also suitable for providing treatment recommendations for swiftly advanced HCC cases [12]. Nevertheless, increasing evidence has shown that all these staging possess certain limitations in predicting survival outcomes of advanced HCC [13]. Currently, more attention is paid to explore novel biological indicators to improve the assessment of HCC prognosis [14, 15].

As a highly heterogeneous cancer, the treatment of HCC is similarly challenging [16]. As the standard first-line treatment modality for intermediate stage HCC, transcatheter arterial chemotherapy (TACE) urgently needs to be improved [17]. The systemic therapy of advanced HCC is still limited in traditional chemotherapeutics (e.g., Oxaliplatin, Fluorouracil and Lenvatinib). Despite efforts to improve the efficacy of chemotherapeutics, the overall survival (OS) outcomes of advanced HCC have only seen a marginal increase of 1 year [18]. Due to the limitations of traditional therapies, immunotherapy has entered the clinical practice stage in the treatment of HCC [19]. For example, targeting regulatory T cells has been reported as a promising approach in the immunotherapy of HCC [20, 21]. But in fact, the role of the immune response in HCC still needs to be complemented. T cells, particularly effector or tissue resident T cells, which have been found to be involved in the protection against tumor cells, are often frequently dysfunctional in HCC [22]. Moreover, it is regrettable that as the key initiation mechanism of immune response, the roles of antigen-presenting-cells (APCs) in HCC immunotherapy are also urgently explored.

Currently, multiple molecular characteristics have been reported to be used in adjuvant treatment of HCC, including tumor mutation burden (TMB), immune checkpoint inhibitor (ICI) treatment, molecular drugs, etc. In addition to immunotherapy, TMB could trigger antitumor activity by activating tumor reactive CD39CD8 T cells, suggesting that TMB is also a potential target for immunotherapy [23]. Recent studies have also revealed the feasibility of TMB as a predictor of treatment decisions and clinical outcomes in advanced HCC [24]. ICI treatment targeting anti-programmed cell death-1 (anti-PD-1) or its ligand (anti-PD-L1) plays an important role in improving the OS rate of HCC [25]. It may provide new methods for the management of patients with advanced HCC [26]. Moreover, several molecular drugs have also been used as primary adjuncts in the management of advanced HCC [27]. Both Sorafenib and Lenvatinib are proven to be the most effective systemic drugs with clinical efficacy of HCC [28]. As reported in previous studies, long non-coding RNAs (LncRNAs) have been shown to play a more distinct role in downregulating cancer cell antigen presentation and intronic tumor suppression compared to coding RNAs [29, 30]. Emerging evidence have revealed that, especially in HCC, LncRNAs play a significant role in the regulation of the immune microenvironment, sustained proliferation and invasion [31]. Moreover, an increasing number of studies have also validated LncRNAs as powerful biomarkers with superior pleiotropy to influence cancer phenotypes and may enable precision therapies for HCC [32]. Therefore, when attempting to construct a signature to accurately forecast prognosis through APCs-related genes, LncRNAs provide more advantages in evaluating the tumor progression and survival. Meanwhile, despite the prospect that multiple adjuvants contribute to HCC treatment, the clinical outcomes of HCC patients are currently still not promising. There is an urgent need to develop ideal indicators to benefit the prognostic assessment of HCC, and provide many improvements for the treatment of HCC.

In this study, the combination of APC related genes (ARGs) and T-cells-infiltration (TCI) estimation was used to identify APC-TCI related LncRNAs (ATRLs). Based on the integration of 15 machine learning (ML) algorithms, a novel APC-TCI derived LncRNA signature (ATLS) was constructed using the expression profiles of ATRLs. The predictive performance of ATLS was validated in 3 public datasets and an external clinical cohort. In addition, the superior predictive capacity of ATLS was highlighted by incorporating several vital clinical characteristics and molecular features for comparison. Moreover, the correlations between ATLS and molecular characteristics (e.g., tumor mutation, drug sensitivity, PD-1/PD-L1 and T cells regulators, etc.) were also explored. Taken together, this work contributes to providing a novel biology tool to improve the clinical outcomes and precision treatment of HCC.

## Materials and methods

### Data collection and preparation

A total of 805 HCC patients were enrolled in this study, containing TCGA cohort (*n* = 365), GSE14520 cohort (*n* = 221), GSE76427 cohort (*n* = 115) and The First Affiliated Hospital of Wenzhou Medical University (FAHWMU) cohort (*n* = 104). Among them, the entire RNA expression profiles and clinical characteristics (e.g., age, gender, tumor grade, T stage, N stage and M stage) for patients in the TCGA cohort were obtained in The Cancer Genome Atlas (https://portal.gdc.cancer.gov/). For further analysis, the RNA expression profiles were normalized according to Fragments Per Kilobase of exon model per Million mapped fragments (FPKM) methods. Meanwhile, we matched the clinical characteristics and RNA expression profiles, extolled the unfit patients. Thus, we obtained the TCGA cohort as the training cohort. RNA expression profiles from GSE14520 and GSE76427 cohorts were downloaded from the Gene Expression Omnibus (GEO) database (https://www.ncbi.nlm.nih.gov/geo/). The data were retrieved based on the GPL3921 [HT_HG-U133A] Affymetrix HT Human Genome U133A Array (GSE14520 cohort) and GPL10558 Illumina HumanHT-12 V4.0 expression bead chip (GSE76427 cohort). Their clinical traits, containing age, gender, ALT, main tumor size, multinodular, cirrhosis, TNM stage, BCLC stage, CLIP stage and AFP were also obtained from GEO database. Both GSE14520 cohort and GSE76427 were treated as testing cohort in this study. The IMvigor210 cohort was installed based on the R package “Imvigor210CoreBiologies”.

An additional 104 HCC patients, with complete baseline clinical characteristics and expression files of 7 optimal APC-TCI related LncRNAs (AC073611.1, AL050341.2, LINC02321, LUCAT1, LINC02362, LINC01871, ZNF582-AS), were enrolled from FAHWMU (Wenzhou, China). The expression profiles were obtained based on the quantitative real-time PCR (qRT-PCR). The endogenous expressions of the above 7 LncRNAs in 30 paired HCC and adjacent non-tumorous tissue samples were provided (Fig. S1). It was found that all these LncRNAs were significantly differentially expressed in HCC compared with adjacent non-tumorous tissue samples. The primer sequences of 7 LncRNAs used for qRT-PCR were listed in Table S1. The collection of FAHWMU cohort was reviewed and approved by the human research ethics committee of the FAHWMU. All patients/participants provided their written informed consent to participate in this study. Among the clinical characteristics in the FAHWMU cohort, the laboratory variables were taken from the results of a test closest to the date of surgery, included Hepatitis B, a-fetoprotein (AFP), CEA (carcinoembryonic antigen) and carbohydrate antigen 19–9 (CA199). And the histopathological variables (including Tumor size, Lymph node invasion, Vascular invasion, Perineural invasion and China Liver Cancer (CNLC) stage) were also included based on the professional pathological assessment. Based on the 8^th^ edition of the AJCC Staging Manual, the TNM stage for each HCC patient was obtained. In addition, age and gender were included as the demographic characteristics. Clinical characteristics in the FAHWMU cohort were presented in Table S2. FAHWMU cohort was treated as the testing cohort to further validate the predictive capacity of ATLS. A total of 107 APCs-related genes (ARGs) were obtained from the MsigDB database (http://www.gsea-msigdb.org/) (Table S3).

### qRT-PCR

The total RNA from the liver tissues of the FAHWMU cohort was extracted using TRIzol reagent and then reverse transcribed into cDNA using ribo SCRIPTTM reverse transcription kit. SYBR Green master mix was added, and real-time PCR was carried out using a 7500 rapid quantitative PCR system (Applied Biosystems, USA). The expression levels of LncRNAs were calibrated with glyceraldehyde-3-phosphate dehydrogenase (GAPDH). The CT value of each well was recorded, and the relative levels of LncRNAs were calculated using the 2^−ΔCt^ method.

### Tumor immune infiltration cells infiltration

In this study, CIBERSOFT algorithm was applied to quantify the contents of 22 immune infiltration cells (ICIs) infiltration. CIBERSOFT was an analytical tool from the Alizadeh lab and the Newman lab for estimating gene expression profiles [33]. Gene expression data were used to estimate the abundance of member cell types in mixed cell populations via CIBERSOFT. In addition, single sample gene set enrichment analysis (ssGSEA) was also employed based on R package “GSVA” to calculate the relative infiltration of 12 immune cells and 11 immune pathways. Moreover, the relative infiltration contents of 6 immune cells were obtained from TIMER database (http://timer.cistrome.org/). According to the Estimation of Stromal and Immune cells in Malignant Tumor tissues using Expression data (ESTIMATE) algorithm, the proportion of infiltrating stromal cells and immune cells in each patient with HCC was estimated [34].

### Consensus clustering analysis

Based on the T cells infiltration profiles, consensus clustering was performed by R package “ConsensusClusterPlus” [35]. In the cumulative distribution function (CDF) curves with the consensus index value from 0.1 to 0.9, the k value with the smallest slope was considered as the optimal value to separate the TCI clusters [36].

### Weighted correlation network analysis (WGCNA)

WGCNA analysis, based on the WGCNA package, was used to describe patterns of gene association between different samples [37]. The scale-free network was constructed through the total expression files of TCGA cohort. And according to the clusters of TCI and network, an appropriate soft threshold β was calculated for the co-expression network construction. Then, the dynamic tree cutting approach was employed to conduct the module identification. The modules with the highest correlation were identified by further filtering to identify key module clusters. Finally, we determined the LncRNAs with high gene significance (GS) and module membership (MM) as the significant TCI related LncRNAs.

### Machine learning algorithms integrative

We selected a total of 5 ML algorithms and developed 15 algorithms combinations to construct the ATLS model. The ML algorithms included RandomForest, Stepwise Cox (StepCox), Logistic, SurvivalSVM and Lasso. RandomForest was implemented through the randomForestSRC package. It had two parameters, ntree and mtry. The ntree represents the number of trees in the forest, while the mtry is the number of randomly selected variables for splitting at each node. The Lasso and Logistic regression analyses were performed via the glmnet package. The regularization parameter of Lasso regression, λ, was determined by leave-one-out cross-validation analysis. With the aid of the survival package, the stepwise Cox was implemented. In addition, the survival-SVM was implemented via survivalsvm package. The generation of ATLS could be summarized as follows: 1) Univariate Cox regression analysis screened prognostic ATRLs in the TCGA cohort; 2) 15 ML algorithms combinations were applied to primarily construct the ATLS in the TCGA cohort (training cohort); 3) All of the ATLS were also verified in 3 testing cohorts (GSE14520 cohort, GSE76427 cohort and FAHWMU cohort); 4) the Harrell’s concordance index (C-index) was calculated in all cohorts, and the ML integrative with highest mean C-index value was considered as the optimal integrative to develop the ATLS.

### Drug sensitivity test and tumor mutation analysis

Mutation raw data for HCC patients in the TCGA cohort were downloaded through TCGA database (https://portal.gdc.cancer.gov/). After upstream analysis of whole genome sequencing and whole exome sequencing data using the "matpool" R package, we used "Getsamplesummary" function and "getgenesummary" function to retrieve the sample information and gene information of the data set respectively for somatic mutation analysis [38, 39]. The response of HCC patients to possible chemotherapeutics drugs (including Doxorubicin, Sorafenib, Tipifarnib, Oxaliplatin, Fluorouracil, Lenvatinib) was predicted using Genomics of Drug Sensitivity in Cancer (GDSC; https://www.cancerrxgene.org). The half-maximal inhibitory concentration (IC50) was estimated using the R package 'pRRophetic' [40].

### Immunohistochemistry

Immunohistochemistry (IHC) was performed according to the following procedures. 4 μm thick sections were cut from paraffin-embedded liver cancer tissue. The sections were dewaxed, rehydrated. Then, microwave was used for antigen repair and hydrogen peroxide block was performed to reduce the nonspecific background staining. 10% serum was used to seal the sections for 1 h at 37℃. Then, the sections were incubated with anti PD-1 or PD-L1 antibody overnight. The next day, the samples were incubated with secondary antibody. Then, DAB detection system was used for the detection of immunoreactive signals.

## Results

### Identification of APC-TCI derived LncRNAs

**Fig. 1:**
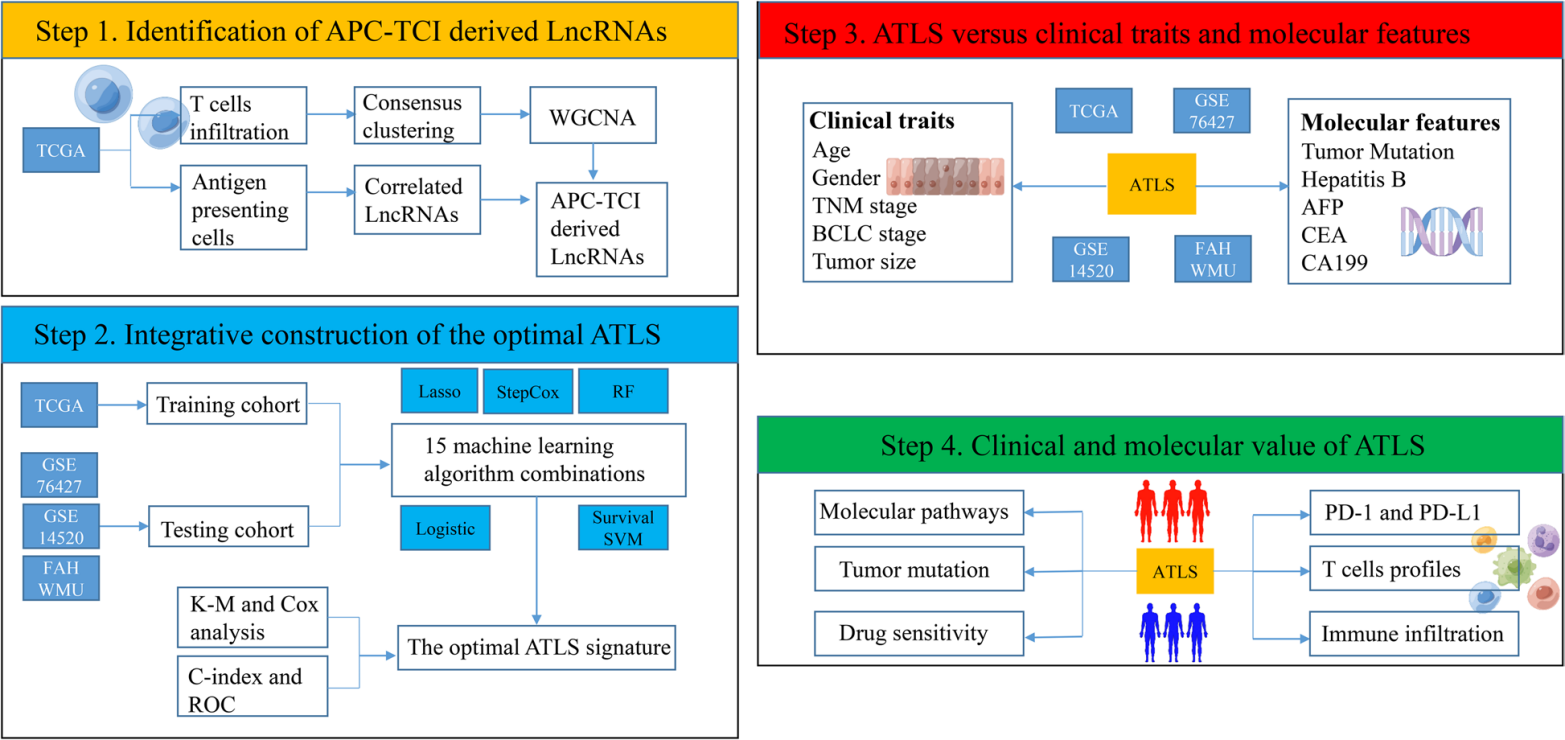
The overall design of this study

As shown in Fig. 1, the overall design of this study could be classified as the following 4 steps: 1) Identification of APC-TCI derived LncRNAs; 2) Integrative construction of the optimal ATLS; 3) ATLS versus clinical traits and molecular features; 4) Clinical and molecular value of ATLS.

**Fig. 2:**
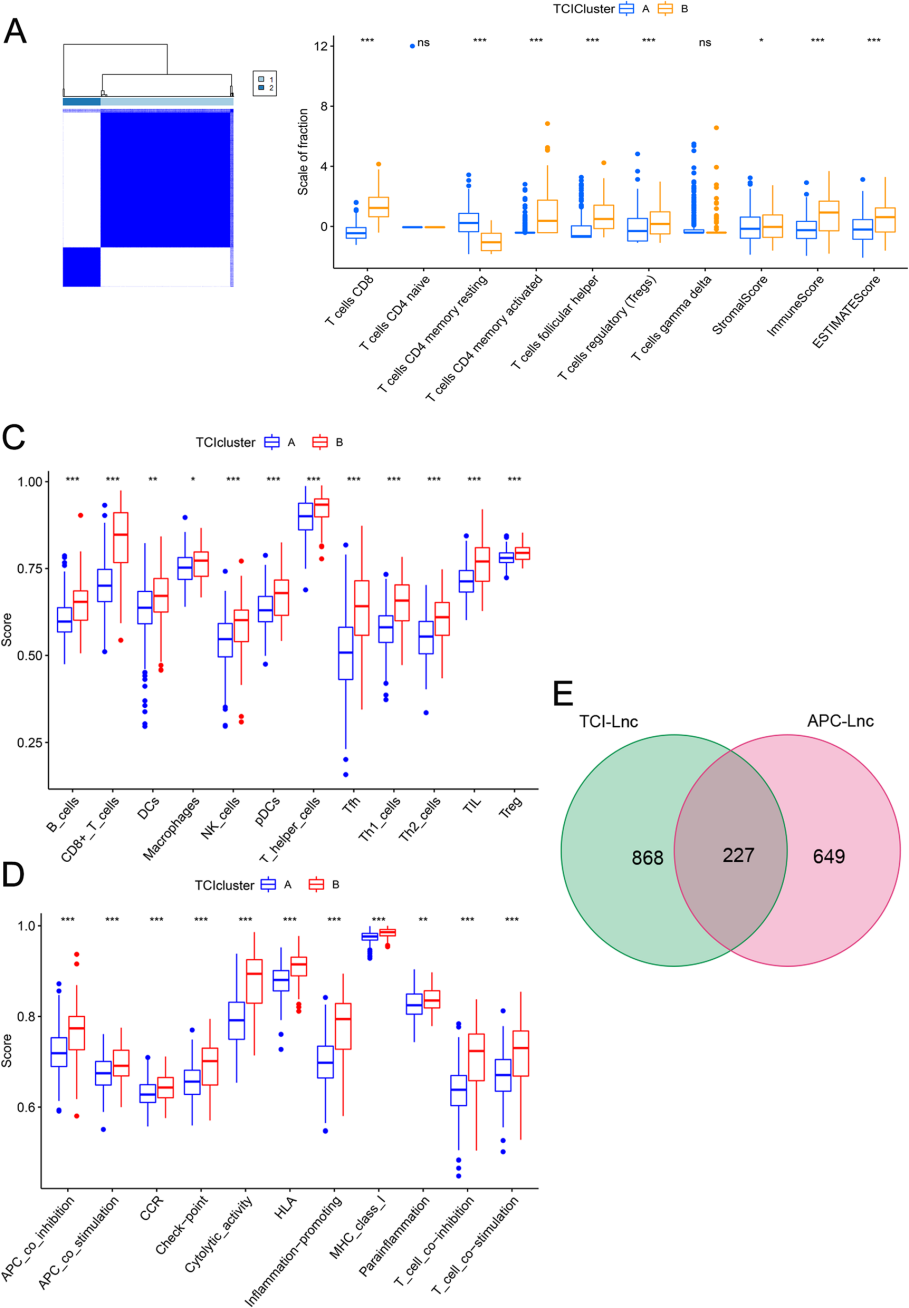
Identification of APCs and TCI derived LncRNAs. **A** The consensus clustering matrix of TCI when k = 2. **B** The distribution of different 7 T cells fractions (based on the CIBERSOFT algorithm) and ESTIMATE scores (based on the ESTIMATE algorithm) between TCI clusters (*: *p* < 0.05, ***: *p* < 0.001). **C** and** D** The distribution of 12 immune cells (C) and 11 immune pathways (D) inferred by ssGSEA algorithm between TCI clusters (*: *p* < 0.05, **: *p* < 0.01, ***: *p* < 0.001). **E** The venn diagram showed the common genes between APCs related LncRNAs and TCI derived LncRNAs (inferred by WGCNA correlation analysis)

Based on the CIBERSOFT algorithm, the relative contents of TCIs were calculated to perform the consensus clustering analysis. By selecting the optimal k value in the CDF curve, key TCI related clusters were determined (Fig. 2A and Fig. S3A). The distribution of T cells contents and ESTIMATE scores was shown in Fig. 2B. It was found that the relative fraction of T cells CD8, T cells CD4 memory activated, T cells follicular helper and T cells regulatory (Tregs) was significantly higher in TCI cluster B than that in TCI cluster A, suggesting a higher T cell reactive activity in TCI cluster B. Moreover, Stromal score, Immune score and ESTIMATE score were also higher in TCI cluster B, indicating that TCI cluster B possessed the higher tumor purity. According to 12 immune cells and 11 immune pathways assessed by ssGSEA, we further verified the robust and extensiveness of TCI clusters. The results indicated that the relative levels of all immune cells (e.g. B cells, CD8 + T cells, DCs, Macrophages, NK cells, pDCs, T helper cells, Tfh, Th1 cells, Th2 cells, TIL, Treg) and immune pathways (e.g. APC co-inhibition, APC co-stimulation, CCR, Check point, Cytolytic activity, HLA, Inflammation-promoting, MHC class I, Parainflammation, T cell co-inhibition, T cell co-stimulation) were obviously lower in TCI cluster A, which was similar to the results above (Fig. 2C and D). In the WGCNA analysis between TCI clusters and LncRNAs, we set the soft threshold β to 5 to obtain the optimal value for co-expression network construction (Fig. S2A and B). Thus, 4 modules were identified, a total of 1095 TCI derived LncRNAs were screened, and the ME brown was considered as the representative of the module (Fig. S3B). Moreover, the association between modules and other clinical characteristics (e.g., age, gender, tumor grade, T stage, N stage, M stage) was also analyzed. With the aid of the Pearson correlation analysis between APC related genes and LncRNAs, a total of 876 APC related LncRNAs were identified. The venn diagram further determined 227 ATRLs (Fig. 2E).

### Integrative construction of APC-TCI derived LncRNA signatures (ATLS) based on the machine learning algorithms

**Fig. 3:**
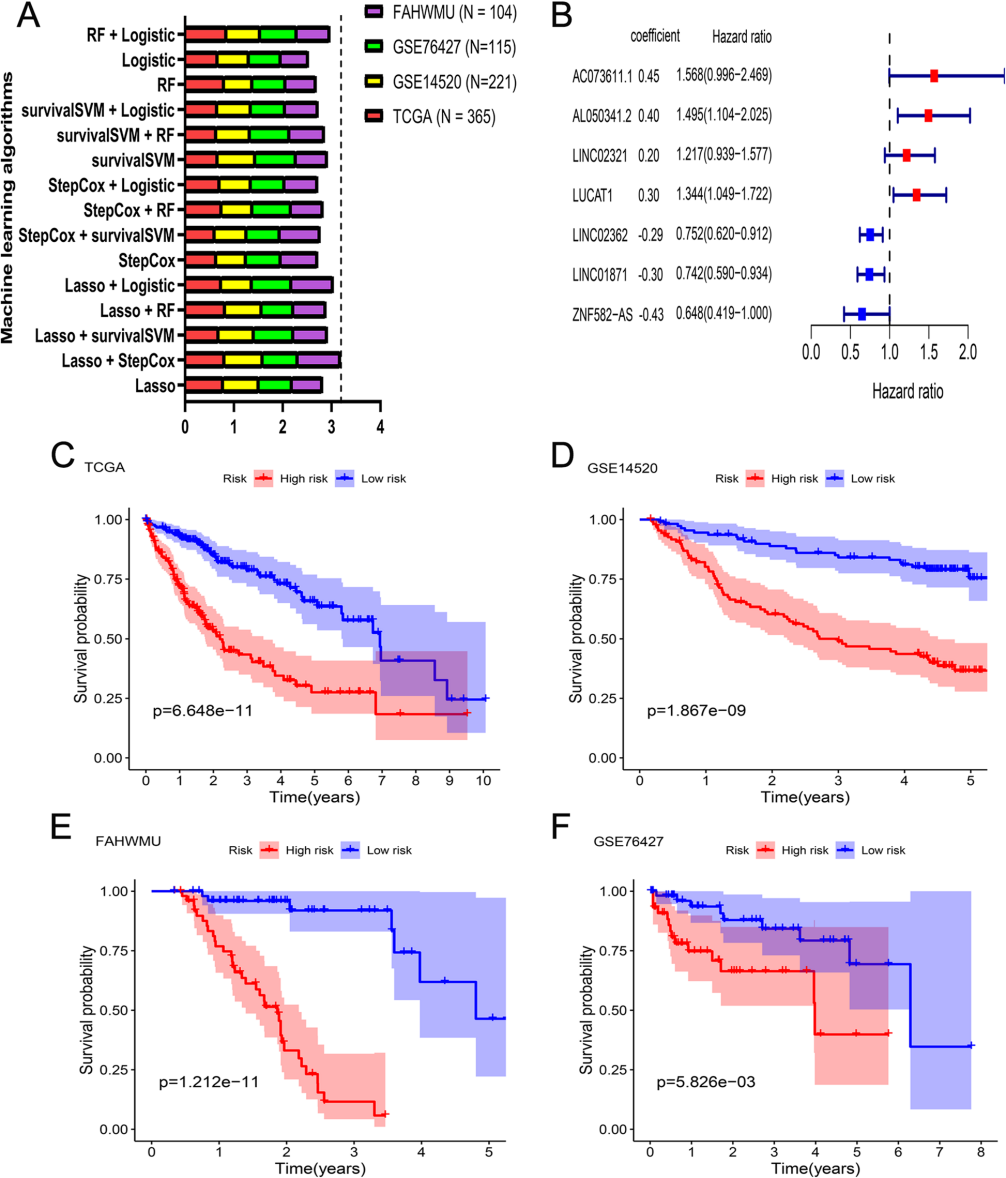
A novel ATLS was developed and primarily validated in all datasets. **A** The comparison of C-index of 15 ML algorithms across training cohort (TCGA cohort) and 3 validation cohorts (GSE14520 cohort, GSE76427 cohort and FAHWMU cohort). **B** The coefficients of 7 LncRNAs finally obtained in the stepCox regression analysis. **C-F** Kaplan–Meier curves of OS according to the ATLS in TCGA cohort (log-rank test: *p* = 6.648e-11) (**C**), GSE14520 cohort (*p* = 1.867e-09) (**D**), FAHWMU cohort (*p* = 1.212e-11) (**E**), and GSE76427 cohort (*p* = 5.826e-03) (**F**)

Based on the expression profiles of 227 ATRLs and the related-OS as well as OS status, a total of 50 prognosis-related ATRLs were screened by applying univariate Cox regression analysis. Theses ATRLs were subjected to our ML algorithms to develop the optimal ATLS. A total of six regression analyses (e.g., Lasso, StepCox, RandomForest, SurvivalSVM, Logistic) were obtained to fit 15 kinds of ML algorithms. By applying these algorithms, we fitted 15 kinds of ML based models. The C-index of each model was calculated in the TCGA cohort and 3 validation cohorts (GSE14520 cohort, GSE76427 cohort and FAHWMU cohort) (Table S4). The mean C-index of 15 prognostic models in all independent cohorts was subsequently calculated to further compare the robust and accuracy for these models. Interestingly, the combination of Lasso and StepCox got the highest average C-index among others (Mean C-index = 0.7675), and the single C-index in the TCGA cohort (0.82), GSE14520 cohort (0.78) and FAHWMU cohort (0.87) was obviously higher than other ones (Fig. 3A). This result could reveal that the combination of Lasso and StepCox was more robust than other ML algorithms integrations. Thus, the combination of Lasso and StepCox was determined as the most powerful integration to construct the optimal ATLS. In the Lasso regression analysis, when the partial likelihood deviation reached the minimum value, the optimal solution was obtained (Fig. S4A and B). Thus, a total of 19 key genes were identified as the critical genes to further perform the StepCox analysis, which finally determined 7 optimal ATRLs (AC073611.1, AL050341.2, LINC02321, LUCAT1, LINC02362, LINC01871, ZNF582-AS) and their coefficients to generate the ATLS (Fig. 3B). The ATLS risk score for each HCC patient was calculated based on the following formula:

ATLS risk score = 0.45*AC073611.1 + 0.40*AL050341.2 + 0.20*LINC02321 + 0.30*LUCAT1 – 0.29*LINC02362 – 0.30*LINC01871 – 0.43*ZNF582-AS.

All patients were assigned into the high- and low-risk groups based on the median value of risk score (Fig. S5). As shown in Fig. 3C-F, patients in the high-risk group had a significantly dismal OS rate in comparison with low-risk patients in the TCGA cohort and 3 validation cohorts (all *p* < 0.05).

### Evaluation of ATLS with clinical traits and molecular features

**Fig. 4:**
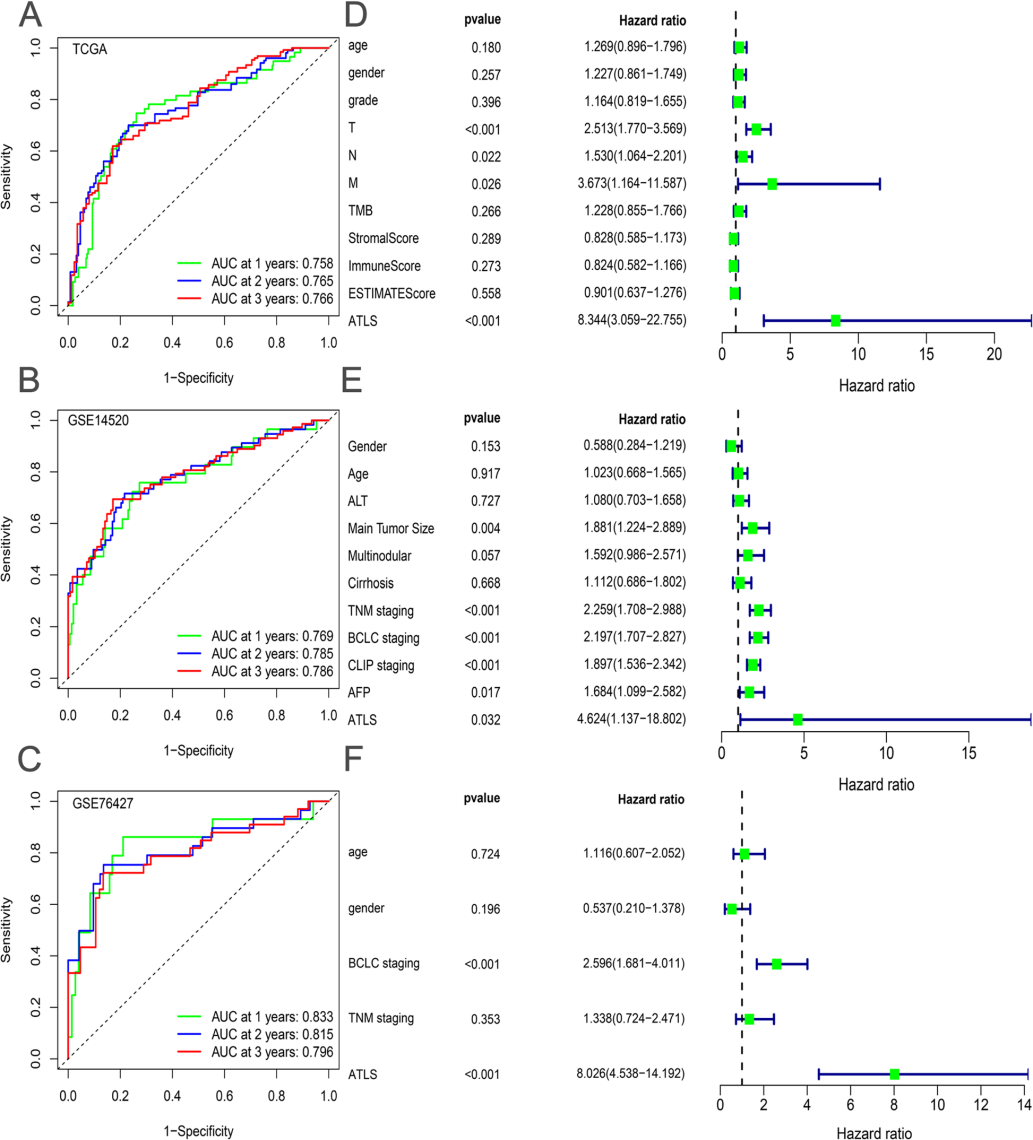
Comparison of ATLS and other clinical characteristics. **A-C** The time-dependent ROC curves of ATLS in the TCGA cohort (A), GSE14520 cohort (**B**) and GSE76427 cohort (**C**) for predicting the OS in the 1^st^, 2^nd^ and 3^rd^ years. **D-F** The univariate Cox analysis of clinical characteristics and ATLS in the TCGA cohort (**D**), GSE14520 cohort (**E**) and GSE76427 cohort (**F**)

Subsequently, we compared the ATLS with other clinical traits in all independent cohorts. The results were analyzed by time dependent ROC curves and univariate Cox analysis. As indicated by Fig. 4A, the area under the ROC curve (AUC) value of ATLS reached 0.758 in the 1^st^ years, 0.765 in the 2^nd^ years and 0.766 in the 3^rd^ years in the TCGA cohort. Compared to other clinical traits and molecular characteristics (age, gender, grade, T stage, N stage, M stage, TMB and ESTIMATE scores), the clinical prognostic value of ATLS was significantly better (Fig. 4D, HR = 0.834, 95%CI = 3.059–22.755). In the GSE14520 cohort and GSE76427 cohort, the AUC value of ATLS reached 0.75, which further verified the robust performance of ATLS (Fig. 4B and C). In addition, the potential value of ATLS as independent prognostic indicator was further validated in the GSE14520 cohort (Fig. 4E, HR = 4.624, 95%CI = 1.137–18.802) and the GSE76427 cohort (Fig. 4F, HR = 8.026, 95%CI = 4.538–14.192).

### Validation of ATLS in the external clinical cohort

**Fig. 5:**
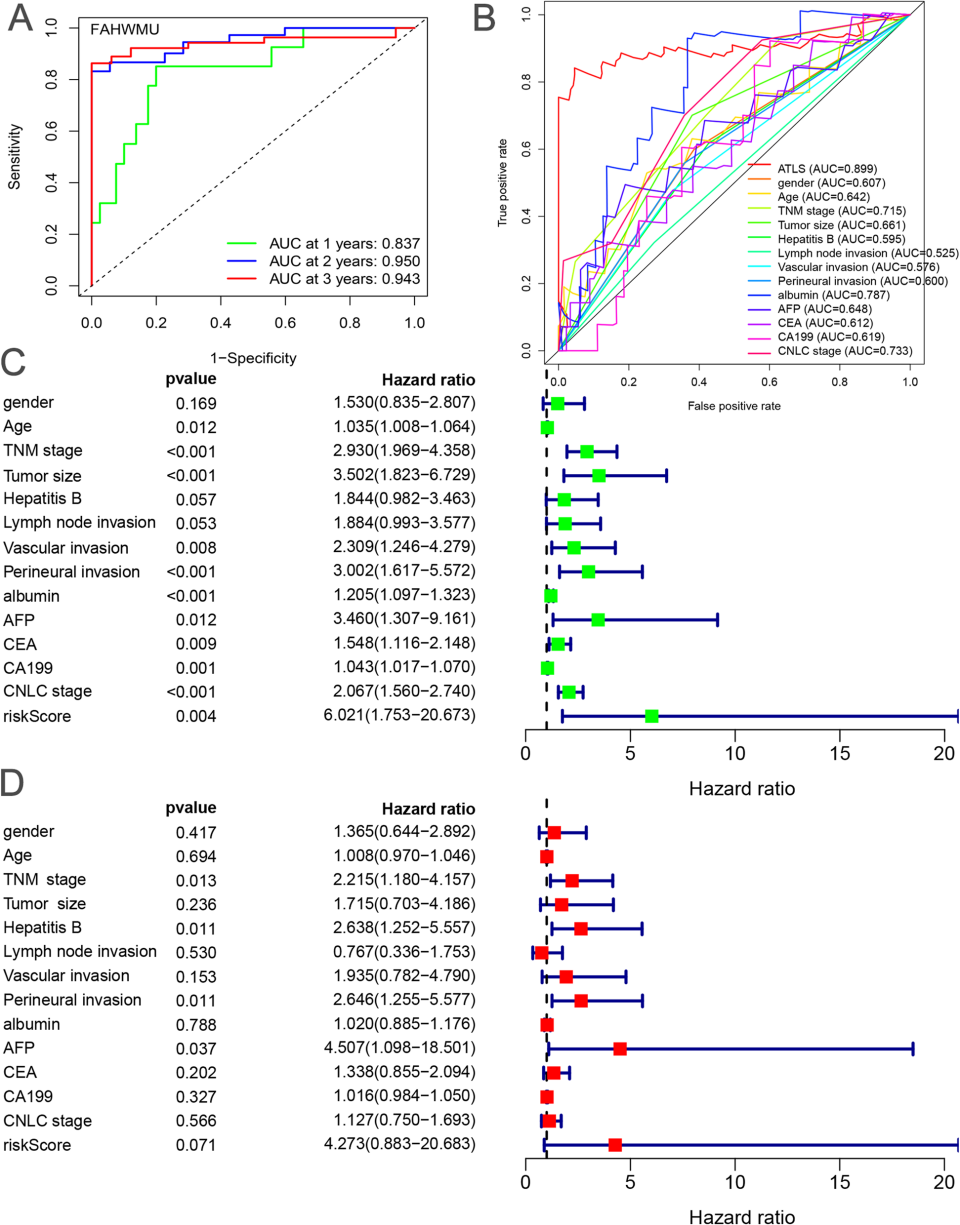
ATLS could serve as an independent risk factor. **A** Time dependent ROC curves for predicting the OS in the 1^st^, 2^nd^ and 3^rd^ years of ATLS in the FAHWMU cohort. **B** The comparison of ATLS and other external clinical traits (e.g., gender, age, TNM stage, CNLC stage, Tumor size, Hepatitis B, Lymph node invasion, Perineural invasion, albumin, AFP, CEA and CA199) in predicting the OS for the patients in the FAHWMU cohort. **C** Univariate Cox analysis displayed the individual prognostic value of clinical traits and ATLS. **D** Multiple Cox analysis showed the combined prognostic value of clinical traits and ATLS

In the FAHWMU cohort, it was found that the AUC values of ATLS were 0.837, 0.950, 0.943 in the 1^st^, 2^nd^ and 3^rd^ years, respectively (Fig. 5A). The multivariate ROC curves showed that the average AUC value of ATLS (AUC = 0.899) was also significantly higher when compared with other clinical features and molecular traits (e.g., gender, age, TNM stage, CNLC stage, Tumor size, Hepatitis B, Lymph node invasion, Vascular invasion, Perineural invasion, albumin, AFP, CEA and CA199) (Fig. 5B). The univariate Cox analysis showed that several clinical traits (age, TNM stage, CNLC stage, Tumor size Vascular invasion, Perineural invasion, albumin, AFP, CEA and CA199) and ATLS could independently predict the prognosis as independent indicators (Fig. 5C, all *p* < 0.05, HR > 1, ATLS: *p* = 0.004, HR = 6.021, 95%CI = 1.753–20.673). The multivariate Cox regression analysis further revealed that the ATLS could serve as an independent risk indicator compared to other clinical traits (Fig. 5D, *p* = 0.071, HR = 4.273, 95%CI = 0.883–20.683).

### The implication of ATLS to the molecular characteristics and immune infiltration

**Fig. 6:**
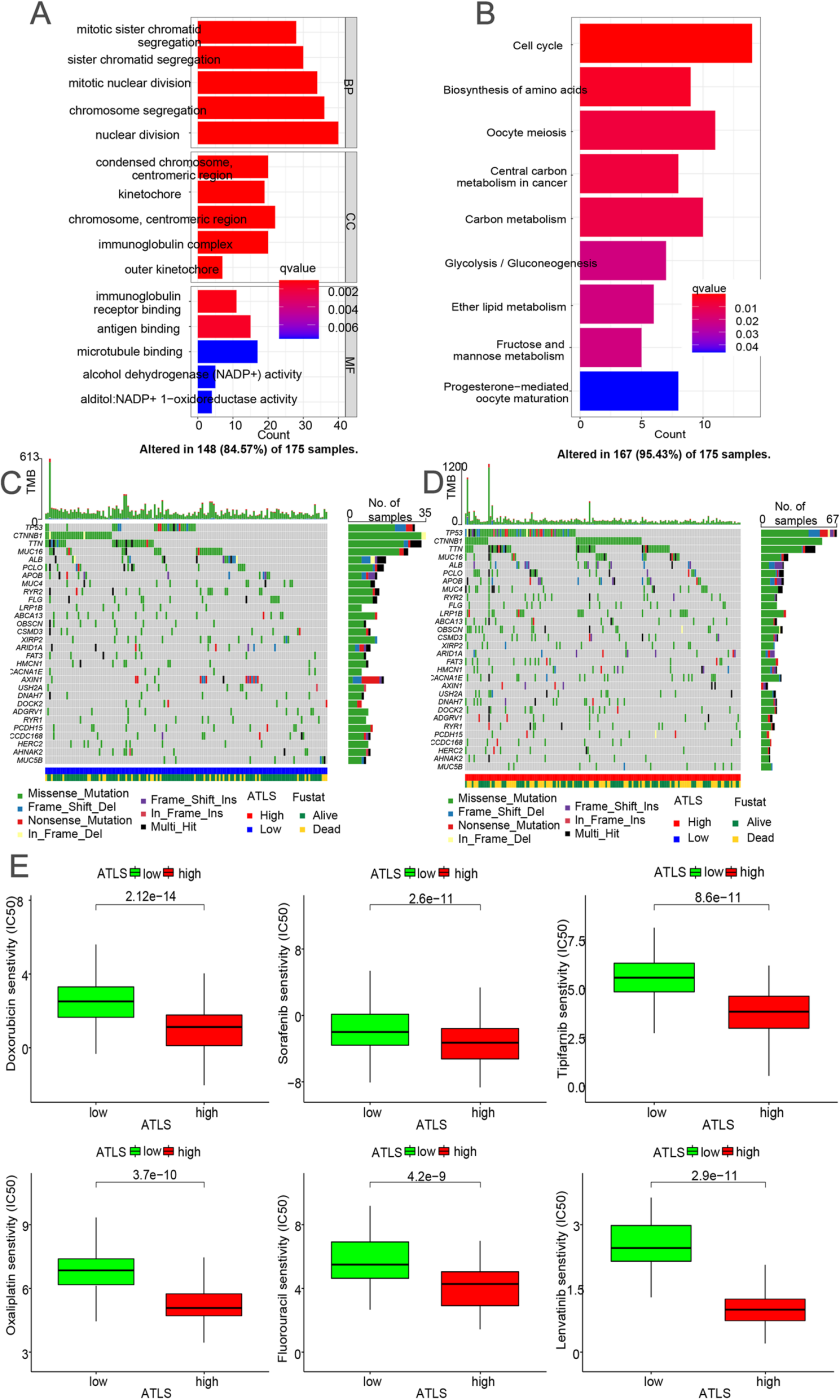
The influence of ATLS to the molecular characteristics. **A** GO function enrichment analysis based on the DEGs selected by ATLS. **B** KEGG pathways enrichment analysis of ATLS. **C** TMB characteristics for individual HCC patient with low ATLS score. **D** TMB characteristics for HCC patients in the high ATLS group. **E** Drug sensitivity analysis displayed the sensitivity of 6 chemotherapeutics drugs (Doxorubicin, Sorafenib, Tipifarnib, Oxaliplatin, Fluorouracil, Lenvatinib) to different ATLS groups

Lastly, the GO and KEGG enrichment analyses were performed to explore the molecular mechanism of the ATLS (Fig. 6A) [41, 42]. Our results demonstrated that the cellular functions between ATLS groups were mainly differed from key regulatory role like cell proliferation and immune response (e.g., nuclear division, chromosome segregation, immunoglobulin receptor binding, immunoglobulin complex, etc.). Similarly, the cellular pathways were mainly differed form cell cycle and important metabolic pathways (e.g., Central carbon metabolism in cancer, Cell cycle, Carbon metabolism, etc.) (Fig. 6B). The distribution of mutant gene, mutation frequency and mutation type for HCC patients in the low ATLS group was shown in Fig. 6C. We also found that the relative levels of mutation frequency and TMB were significantly lower in the low ATLS group (Fig. 6D). In addition, the ATLS was also positively correlated with OS of HCC patients. In Fig. 6E, the sensitivity of patients in ATLS groups to 6 chemotherapeutics drugs (Doxorubicin, Sorafenib, Tipifarnib, Oxaliplatin, Fluorouracil, Lenvatinib) was evaluated. It was found that patients with high ATLS score tended to have lower IC50s for these chemotherapeutics drugs (all *p* < 0.05), illustrating that they may have higher sensitivity to chemotherapy treatment.

**Fig. 7:**
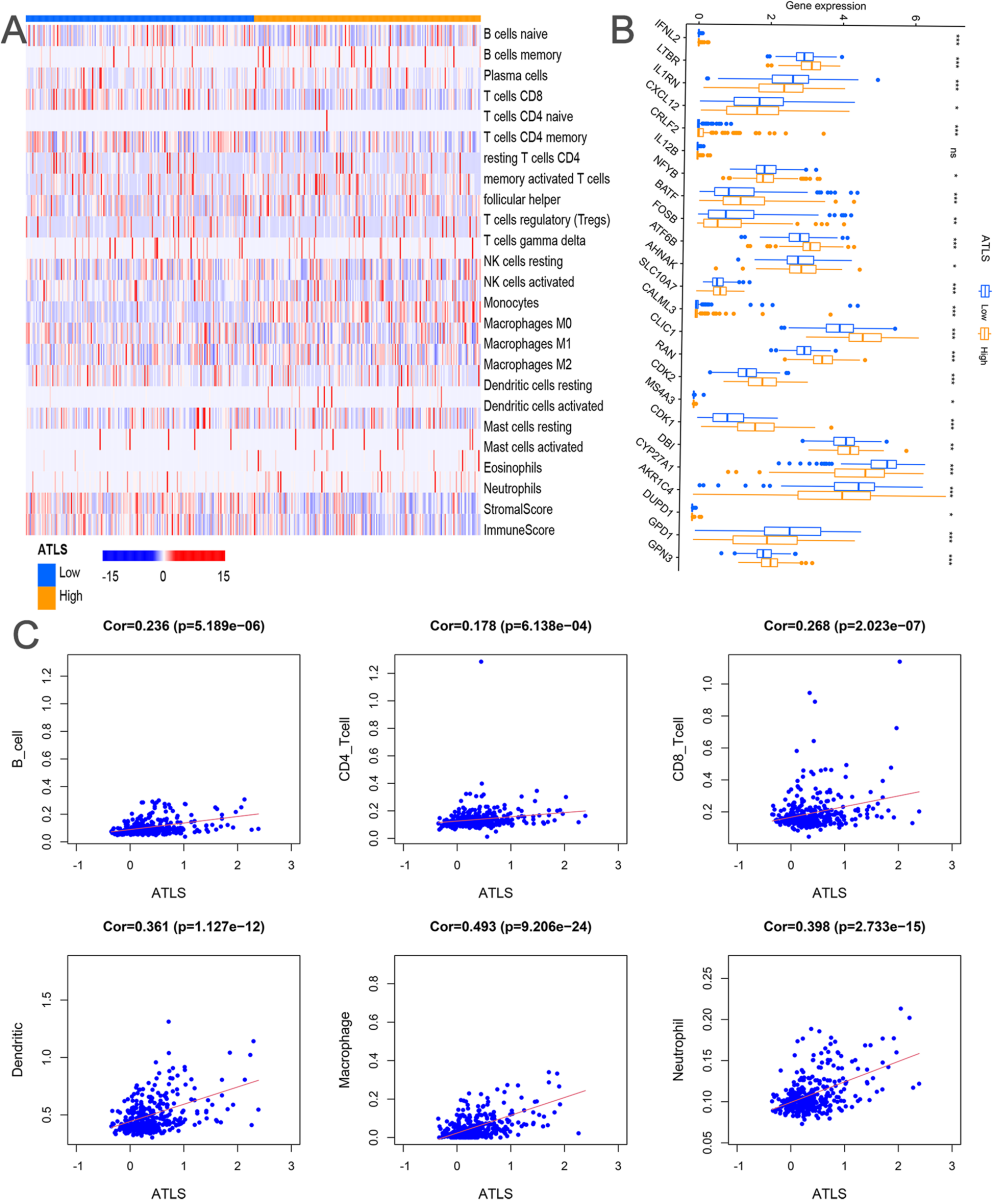
The implication of ATLS to immune infiltration. **A** The distribution of ATLS and 22 main tumor immune infiltration cells in the TCGA cohort.** B** The boxplot displayed the association between ATLS and T cells profiles derived genes in the TCGA cohort (*: *p* < 0.05; **: *p* < 0.01; ***: *p* < 0.001). **C** The scatterplots demonstrated the correlation between ATLS and immune infiltration cells

As shown in Fig. 7A, the distribution of 22 main tumor immune infiltration cells and ESTIMATE scores between different ATLS groups was described. Figure 7B revealed that ATLS could significantly assess the levels of T cell regulator factors (all *p* < 0.05). The association between ATLS and PD-1/PD-L1 expression levels was analyzed based on the IHC staining images (Fig. S6A-D). It was found that the expression levels of PD-1/PD-L1 were remarkably higher in the high-risk group. Through the immune cells data obtained from TIMER database, we further validated the positive correlation between ATLS and immune infiltration (Fig. 7C, all *p* < 0.05). As shown in Fig. S7, it was demonstrated that patients with higher ATLS score had a higher response to anti-PD-L1 treatment (IMvigor210 cohort, *p* = 0.007).

## Discussion

The poor prognosis of HCC may be mainly attributed to the failure of early detection and early diagnosis in most patients [43]. Current HCC staging systems (e.g., TNM staging system, JSH staging system, BCLC staging system, etc.) have provided great convenience in the prognostic evaluation and treatment regimen qualification of HCC [44, 45]. However, due to the complex mechanisms and geographic variations of HCC, these staging systems also have some limitations in the diagnosis and decision-making process for advanced HCC. For example, the TNM staging system lacks measures of liver function and patient physical status, resulting in its poor ability to guide the treatment of patients with newly diagnosed HCC [46]. Currently, there are a large number of studies aiming to explore more robust prognostic indicators for HCC [47, 48]. It has been reported that Circ-ZEB1 may serve as a key indicator for the evaluation of proliferation and apoptosis levels in HCC [49]. CD151 or combination of CD151/c-Met has also been found to play an important role in predicting the invasiveness and prognosis of HCC [50]. It is undeniable that these indicators are greatly beneficial for the assessment of HCC. But as a single gene or pathway, it is difficult to guarantee that no errors are generated in practical applications. Meanwhile, the AUC value of single indicator is less satisfactory when it is used in large cohorts. For these aspects, our ATLS model contributes to complementing the original gaps. With the aid of ML integrations, it is confirmed that the calculation of the ATLS score was straightforward and practical. Considering the possible error from a single index, the ATLS score is more precise and credible in clinical applications. Additionally, as highlighted in our results, ATLS showed a more significant prediction of clinical outcomes compared with traditional staging systems.

It has been reported that increased infiltration of T cells could enhance antitumor immunity and increase the sensitivity of anti-PD-L1 immunotherapy, resulting in the satisfy outcomes for HCC patients [51, 52]. Previously, TCI-related pathways have also been reported to play a key role in the proliferation, migration and invasion of HCC [53]. Targeting these immune-related genes or pathways, many relevant genetic signatures have been constructed to assess their values in the prognosis stratification of HCC [54, 55]. For instance, Ma et al. constructed a signature associated with Jab1/CSN5 in predicting the prognosis of HCC [56]. Huang et al. developed a novel TME related LncRNAs signature to determine the prognosis and immune response of HCC [57]. Recently, Chaudhary et al. also found that multi omics combined features with the aid of ML can effectively predict the prognosis of HCC [57]. All these signatures remarkably benefit the prognostic evaluation of HCC. But unfortunately, the lack of sufficient clinical external cohorts and cross-sectional comparisons inevitably result in the limitations. To ameliorate it, our ML integrations were developed to ensure the precision and efficiency. Thus, our ATLS model has a superior predictive capacity compared with traditional clinical characteristics and molecular features.

Previously, immunotherapy has been proven to be effective in all stages of HCC and may serve as a completely new modality for HCC management [58]. ICI treatment, as breakthrough immunotherapies, also greatly improves the prognosis of patients with advanced HCC [59]. Current studies have revealed that HCC patients with increased PD-L1 expression are more effectively treated with anti-PD-1 and anti-PD-L1 therapies [60]. Our results demonstrated that there was a positive correlation between PD-1/PD-L1 and ATLS. Hence, the ATLS may play a role in assessing ICI treatment. For HCC patients, TP53 mutations are generally associated with poor prognosis [61]. As one of the highly mutated genes, TTN has also been confirmed as a key regulatory gene driving HCC [62]. We found that the ATLS model can effectively predict the mutation levels of TP53 and TTN, which may be a novel indicator in the evaluation of tumor mutation and prognosis of HCC. Doxorubicin has been proven to significantly improve the OS and RFS for HCC patients plus sorafenib [63]. Sorafenib, as a tolerated molecular agent, also has a potential role in the adjuvant treatment of HCC [64, 65]. As highlighted in our results, the ATLS showed the exciting value in assessing the levels of tumor mutation, drug sensitivities, immune infiltration and T cells regulators. All the data suggest that ATLS has the potential to be a beneficial indicator, thus improving prognosis assessment and clinical-decision making for HCC.

The advantages of the ATLS could be summarized as follows: First of all, we integrated multiple ML algorithms to build the ATLS, ensuring its predictive performance. Secondly, the robustness and superiority of the ATLS were further compared among multiple cohorts with a variety of clinical traits and molecular features. In addition, the deeper clinical applications of ATLS were further explored by integrating multiple molecular characteristics. Meanwhile, many limitations of the ATLS should also be revealed. Firstly, larger scale clinical data are needed to further validate the clinical application values of the ATLS. Secondly, more in-depth in vivo or in vitro experiments are needed to further explore the joint regulatory roles of LncRNAs in our ATLS.

## Conclusion

In conclusion, we developed a robust and powerful signature based on the ML integrations in this study. In multiple independent clinical cohorts, the signature showed more superior performance in predicting OS, compared to other clinical indicators. Furthermore, the ATLS also showed the exciting value in assessing the levels of tumor mutation, drug sensitivities, immune infiltration and T cells regulators. The ATLS could serve as a promising indicator, which contributes to improving the prognosis assessment and clinical-decision making for HCC.

## Supplementary Information

**Additional file 1: Fig. S1. A-G **The endogenous expression data of 7-LncRNAs (AC073611.1, AL050341.2, LINC02321, LUCAT1, LINC02362, LINC01871, ZNF582-AS) in 30 paired HCC and adjacent non-tumorous tissue samples (*: *p* < 0.05; **: *p* < 0.01; ***: *p* < 0.001). **Fig. S2.** WGCNA analysis. **A** Analysis of network topology for different soft-threshold power. The left panel shows the impact of soft-threshold power on the scale-free topology fit index; the right panel displays the impact of soft-threshold power on the mean connectivity. **B **The heatmap revealed the eigengene adjacency of modules. **Fig. S3. A **The CDF curves of consensus clustering for each k values. **B** WGCNA correlation analysis between TCI derived DEGs and clinical traits. **Fig. S4. A and B **In the TCGA cohort (*n* = 365), the optimal λ was obtained when the partial likelihood deviance reached the minimum value. **Fig. S5. A-D **The ranking of patients with increased risk score in each cohort (A: TCGA cohort, B: GSE14520 cohort, C: GSE76427 cohort, D: FAHWMU cohort). The median risk score was considered as the cut-off point to assign patients into high-risk group and low-risk group. **Fig. S6.** **A-D** The IHC staining images showed the correlations between ATLS and the relative expression levels of PD-1 (C and D) and PD-L1 (A and B) in the FAHWMU cohort. **Fig. S7. **The correlation between ATLS score and Anti-PD-L1 response in the IMvigor210 cohort (*p* = 0.007). **Table S1.** The primer sequence of 7-LncRNAs used for qRT-PCR. **Table S2. **The clinical characteristics for HCC patients in the FAHWMU cohort. **Table S3. **The lists for all APCs-related genes used in this study. **Table S4.** The details for 15 kinds of prediction models via machine learning integration (combined Lasso regression, StepCox, survivalSVM, RandomForest and Logistic) and further calculated the C-index of each model across all validation datasets (TCGA cohort, GSE14520 cohort, GSE76427 cohort and FAHWMU cohort).

## Data Availability

Public data used in this work can be acquired from the TCGA database (https://portal.gdc.cancer.gov/) and Gene Expression Omnibus (GEO, http://www.ncbi.nlm.nih.gov/geo/). The other data could be obtained from the corresponding author with valid reasons.

## Abbreviations

HCC: Hepatocellular Carcinoma
TCGA: The Cancer Genome Atlas
LASSO: Least absolute shrinkage and selection operator
FDR: False-discovery rate
OS: Overall survival
AUC: Area under the curve
ROC: Receiver operating characteristic
ssGSEA: Single-sample gene set enrichment analysis
K-M: Kaplan–meier
GO: Gene Ontology
KEGG: Kyoto Encyclopedia of Genes and Genomes
ssGSEA: Single-sample gene set enrichment analysis
CDF: Cumulative distribution function
TME: Tumor microenvironment
