# Supplementary material for: Machine learning integrations develop an antigen-presenting-cells and T-Cells-Infiltration derived LncRNA signature for improving clinical outcomes in hepatocellular carcinoma
Source: BMC Cancer. 2023 Mar 28;23:284. doi: 10.1186/s12885-023-10766-w (PMC10053113; doi:10.1186/s12885-023-10766-w)
Supplement: Supplementary file 1 — Additional file 1: Fig. S1. A-G The endogenous expression data of 7-LncRNAs (AC073611.1, AL050341.2, LINC02321, LUCAT1, LINC02362, LINC01871, ZNF582-AS) in 30 paired HCC and adjacent non-tumorous tissue samples (*: p < 0.05; **: p < 0.01; ***: p < 0.001). Fig. S2. WGCNA analysis. A Analysis of network topology for different soft-threshold power. The left panel shows the impact of soft-threshold power on the scale-free topology fit index; the right panel displays the impact of soft-threshold power on the mean connectivity. B The heatmap revealed the eigengene adjacency of modules. Fig. S3. A The CDF curves of consensus clustering for each k values. B WGCNA correlation analysis between TCI derived DEGs and clinical traits. Fig. S4. A and B In the TCGA cohort (n = 365), the optimal λ was obtained when the partial likelihood deviance reached the minimum value. Fig. S5. A-D The ranking of patients with increased risk score in each cohort (A: TCGA cohort, B: GSE14520 cohort, C: GSE76427 cohort, D: FAHWMU cohort). The median risk score was considered as the cut-off point to assign patients into high-risk group and low-risk group. Fig. S6. A-D The IHC staining images showed the correlations between ATLS and the relative expression levels of PD-1 (C and D) and PD-L1 (A and B) in the FAHWMU cohort. Fig. S7. The correlation between ATLS score and Anti-PD-L1 response in the IMvigor210 cohort (p = 0.007). Table S1. The primer sequence of 7-LncRNAs used for qRT-PCR. Table S2. The clinical characteristics for HCC patients in the FAHWMU cohort. Table S3. The lists for all APCs-related genes used in this study. Table S4. The details for 15 kinds of prediction models via machine learning integration (combined Lasso regression, StepCox, survivalSVM, RandomForest and Logistic) and further calculated the C-index of each model across all validation datasets (TCGA cohort, GSE14520 cohort, GSE76427 cohort and FAHWMU cohort). [file 12885_2023_10766_MOESM1_ESM.docx]

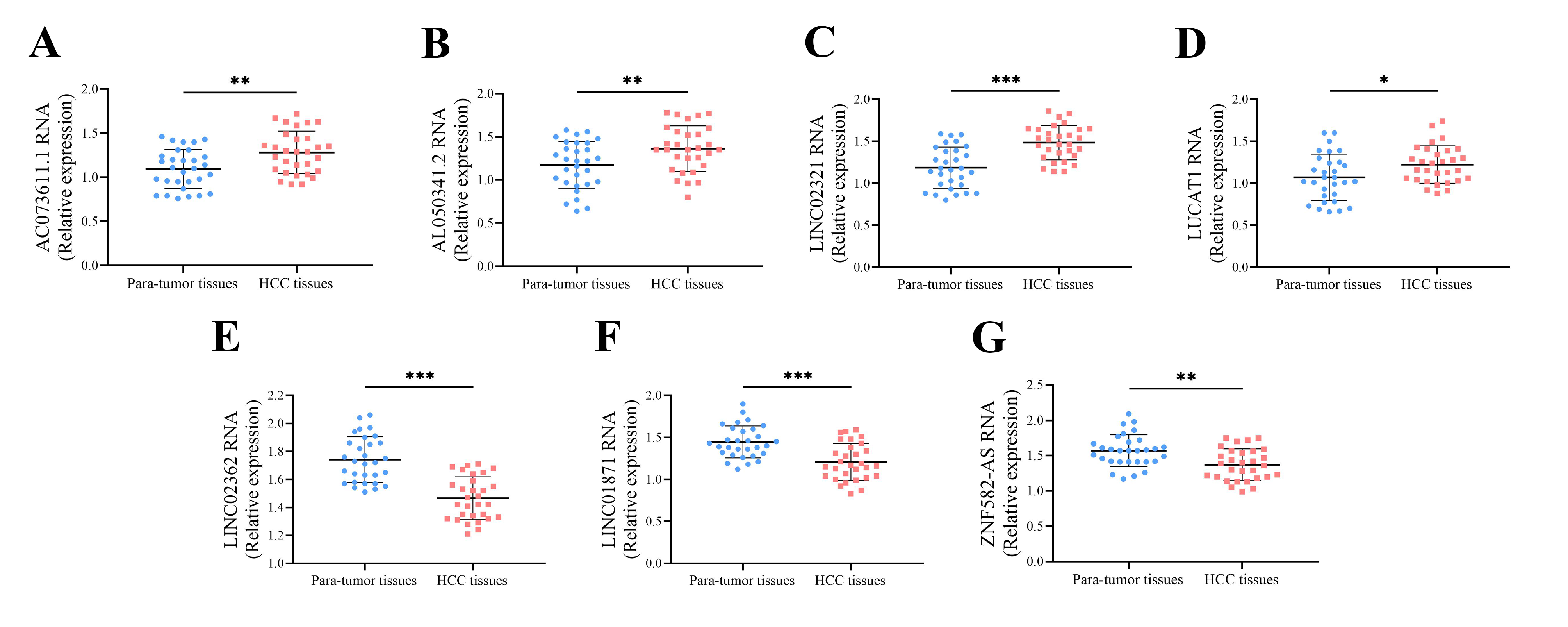
**Supplementary information**

**Fig. S1 A-G** The endogenous expression data of 7-LncRNAs (AC073611.1, AL050341.2, LINC02321, LUCAT1, LINC02362, LINC01871, ZNF582-AS) in 30 paired HCC and adjacent non-tumorous tissue samples (*: p < 0.05; **: p < 0.01; ***: p < 0.001).


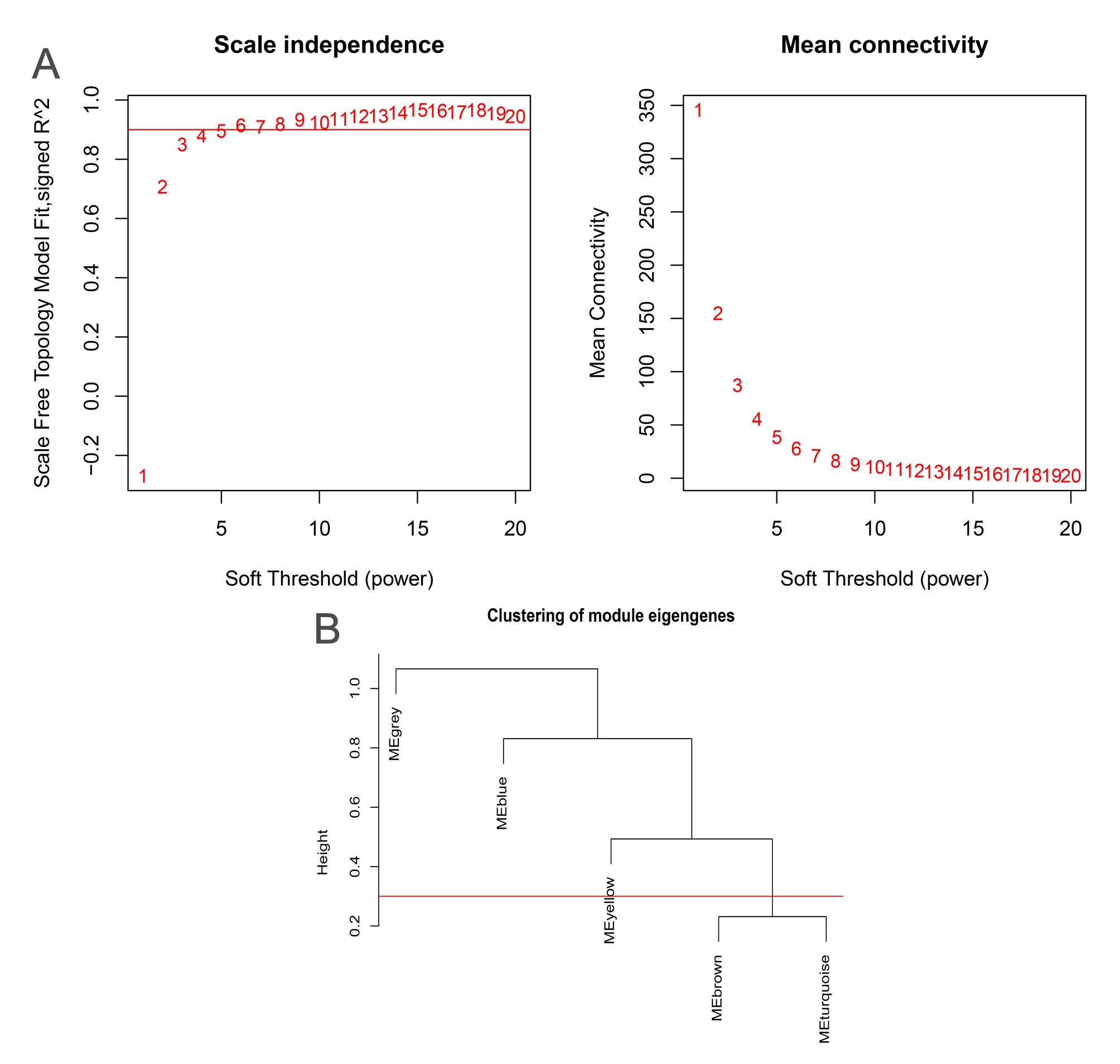


**Fig. S2 WGCNA analysis.** **A** Analysis of network topology for different soft-threshold power. The left panel shows the impact of soft-threshold power on the scale-free topology fit index; the right panel displays the impact of soft-threshold power on the mean connectivity. **B** The heatmap revealed the eigengene adjacency of modules.


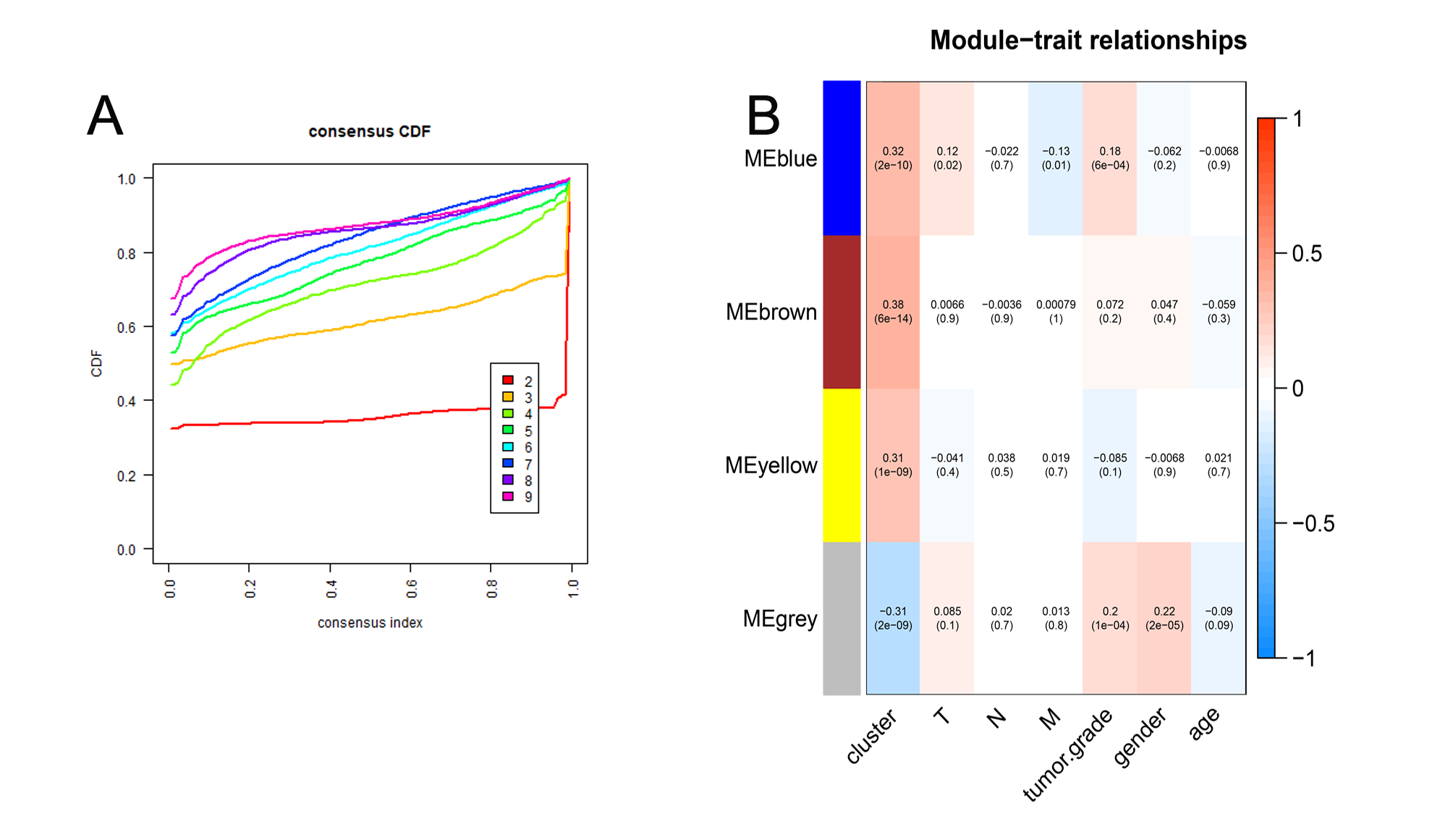
**Fig. S3 A** The CDF curves of consensus clustering for each k values. **B** WGCNA correlation analysis between TCI derived DEGs and clinical traits.


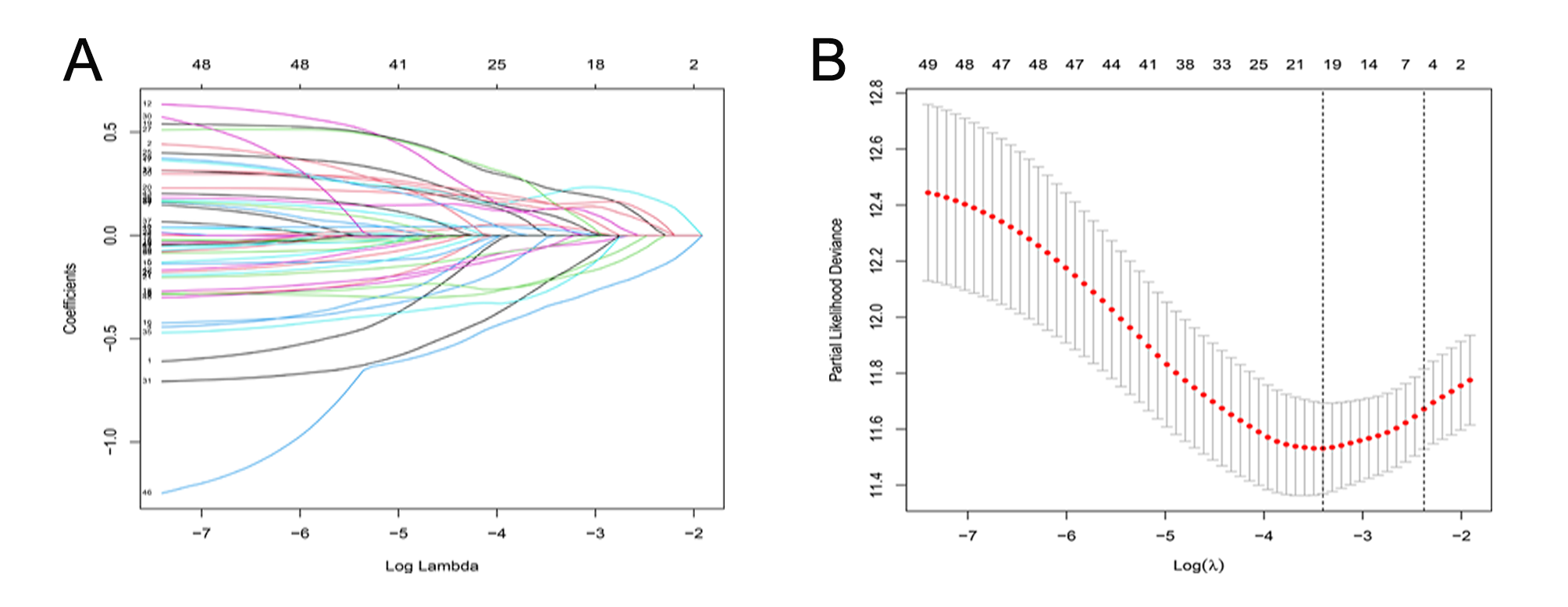


**Fig. S4 A and B** In the TCGA cohort (n = 365), the optimal λ was obtained when the partial likelihood deviance reached the minimum value.


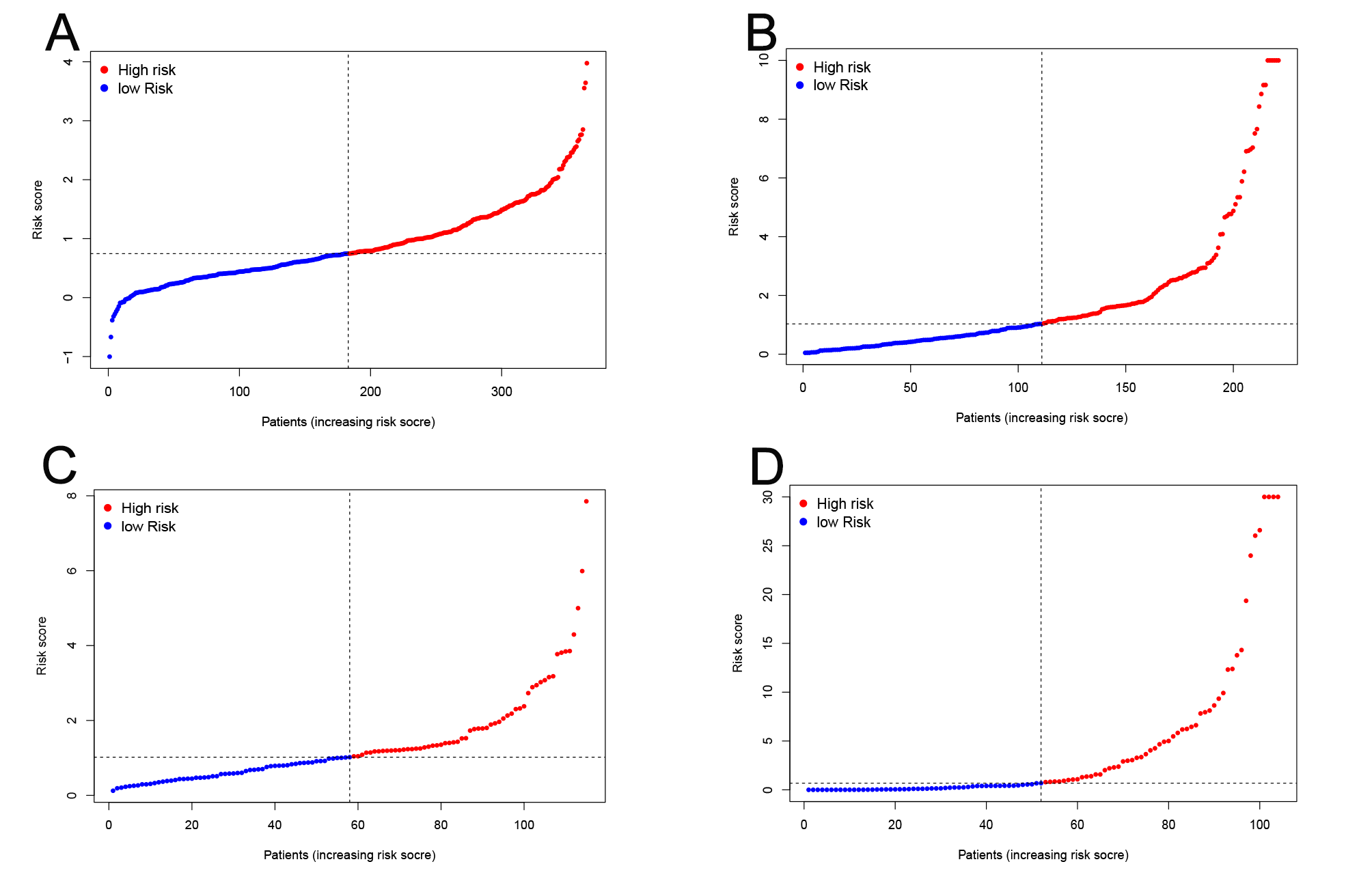


**Fig. S5 A-D** The ranking of patients with increased risk score in each cohort (A: TCGA cohort, B: GSE14520 cohort, C: GSE76427 cohort, D: FAHWMU cohort). The median risk score was considered as the cut-off point to assign patients into high-risk group and low-risk group.

**
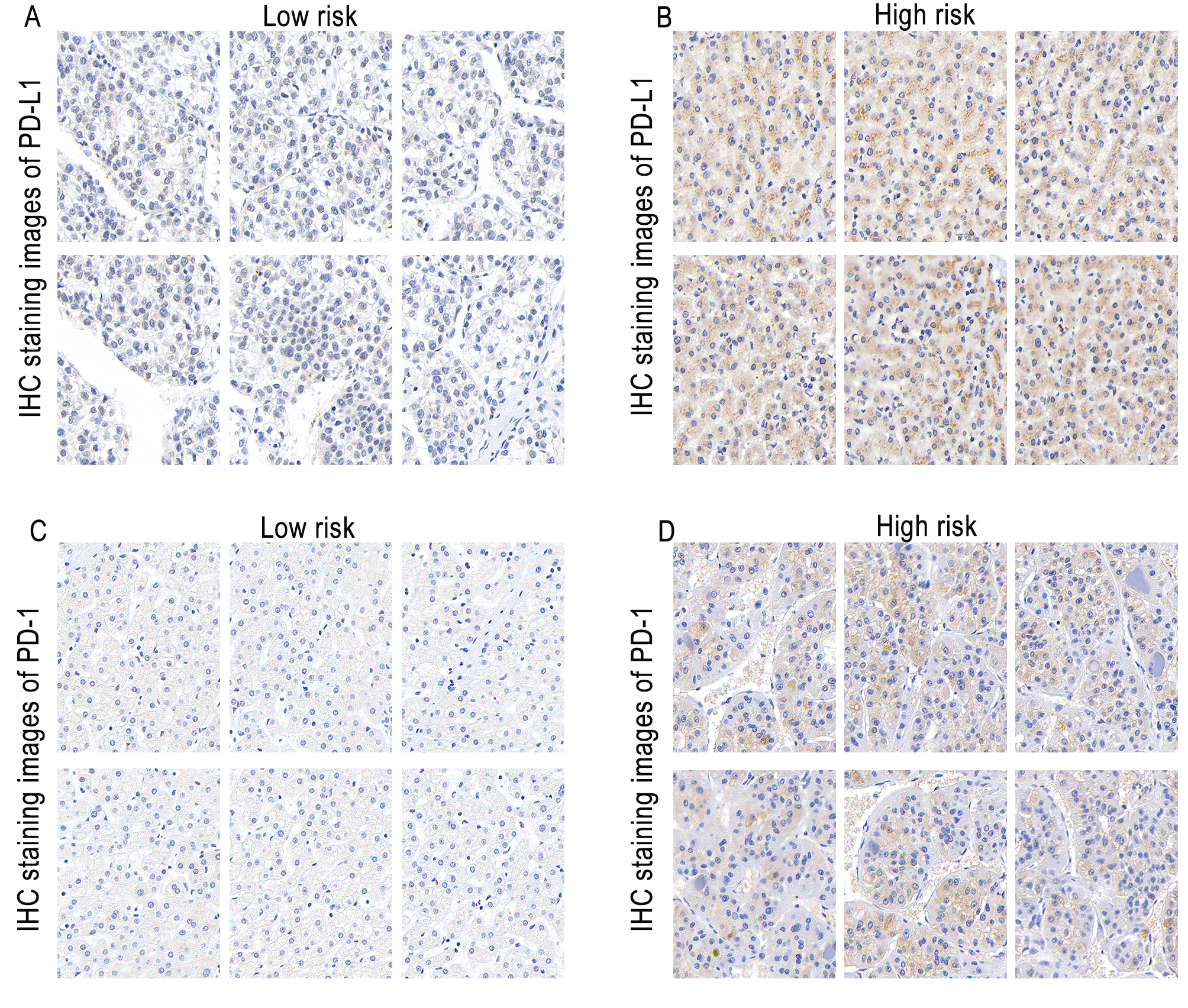
Fig. S6** **A-D** The IHC staining images showed the correlations between ATLS and the relative expression levels of PD-1 (C and D) and PD-L1 (A and B) in the FAHWMU cohort.


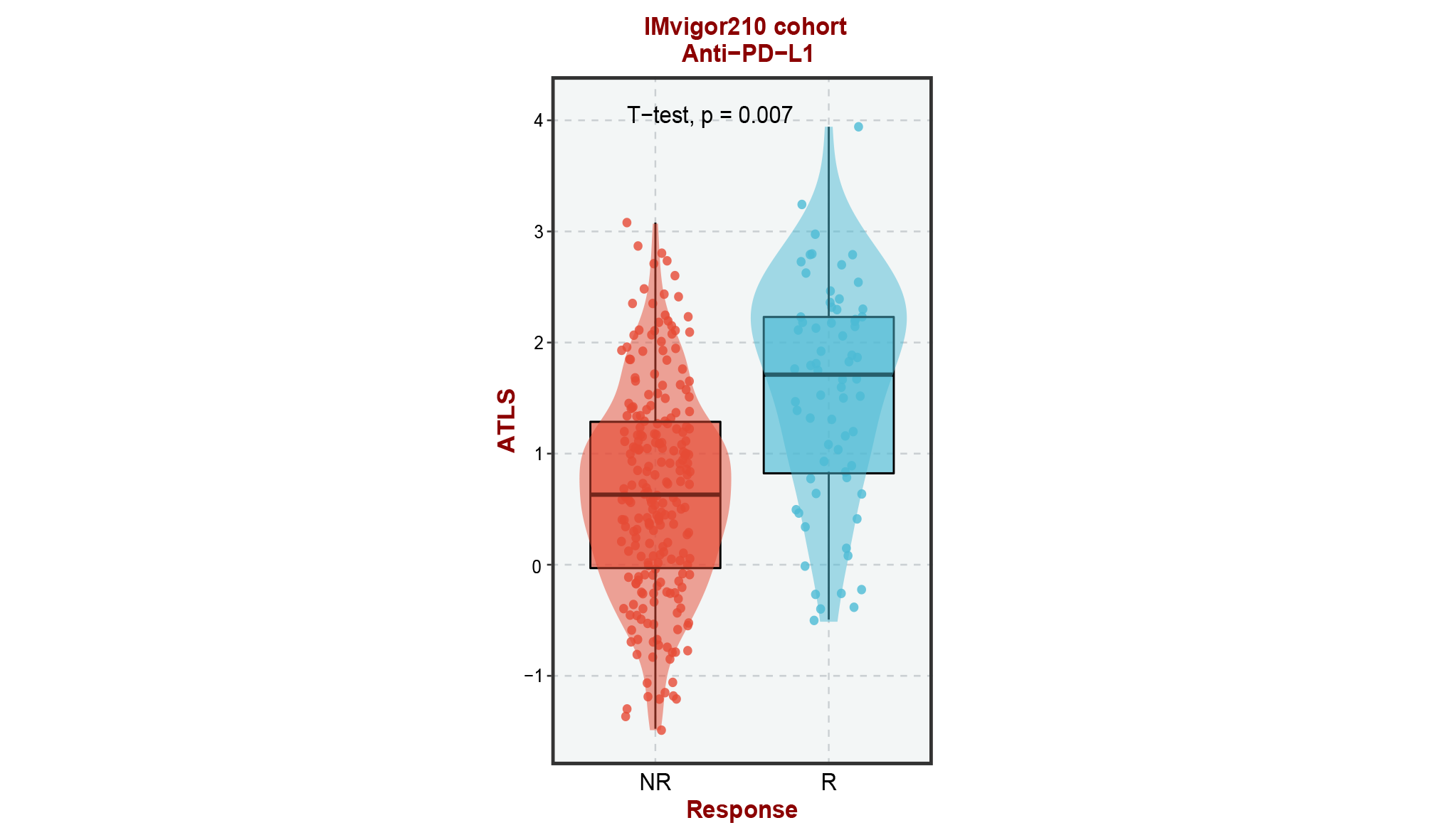


**Fig. S7** The correlation between ATLS score and Anti-PD-L1 response in the IMvigor210 cohort (p = 0.007).

**Table S1** The primer sequence of 7-LncRNAs used for qRT-PCR.

| Gene | Forward (5'-3') | Reverse (5'-3') |
| --- | --- | --- |
| LINC02321 | CTGGTTTTCTCGCTGACCCT | TCAAGAAGGTGGTCGTCAGC |
| LUCAT1 | AAACTGCTAAAGGGGCTGAATG | ATCTTCTGATGGGTTTTGTTCCTT |
| LINC02362 | GTTCTTCACTCTCCACACTCCAA | GCTCAATCAGGTGTGAATGAGGAT |
| LINC01871 | GACGTTAAGCATGCAGCAACT | CTCAGCTTCGGCCTTTGGTA |
| ZNF582-AS | GTTCGTGTGTTCGAGTCCCT | GCTACCACCACACACTGTCA |
| AC073611.1 | CATATCGGTGTCTCCGAGGG | CGCAGGATGCAGAGATTGTG |
| AL050341.2 | GTGCCCTGGAACAAAGCAAG | CAATGTACAGGCGTCCCAGT |
| GAPDH | AAGCCTGCCGGTGACTAAC | CGCCCAATACGACCAAATCAGA |

**Table S2** The clinical characteristics for HCC patients in the FAHWMU cohort.

|  | **FAHWMU cohort (n=104)** |
| --- | --- |
| Age, years | 51.51±11.49 |
| Gender |  |
| male | 49 (58.7%) |
| female | 34 (41.3%) |
| TMN stage |  |
| I | 39 (32.7%) |
| II | 23 (21.2%) |
| III | 17 (38.5%) |
| IV | 8 (7.7%) |
| CNLC stage |  |
| IA | 39 (37.5%) |
| IB | 23 (22.1%) |
| IIA | 24 (23.1%) |
| IIB | 13 (13.5%) |
| IIIA | 5 (4.8%) |
| Tumor size, cm |  |
| ≤5 | 60 (57.7%) |
| >5 | 44 (42.3%) |
| Hepatitis B |  |
| negative | 57 (54.8%) |
| positive | 47 (45.2%) |
| Lymph node invasion |  |
| no | 75 (72.1%) |
| yes | 29 (27.9%) |
| Vascular invasion |  |
| no | 68 (65.4%) |
| yes | 36 (34.6%) |
| Perineural invasion |  |
| no | 64 (61.5%) |
| yes | 40 (38.5%) |
| albumin, g/L | 39.54±3.51 |
| AFP, ng/ml | 2.57±0.31 |
| CEA, ug/L | 1.78±0.84 |
| CA199, U/ml | 47.75±12.85 |

**Table S3** The lists for all APCs-related genes used in this study.

| Number | Gene | Number | Gene | Number | Gene | Number | Gene | Number | Gene |
| --- | --- | --- | --- | --- | --- | --- | --- | --- | --- |
| 1 | ABCB9 | 26 | CTSF | 51 | HLA-DQB1 | 76 | PDIA3 | 101 | THBS1 |
| 2 | ACE | 27 | CTSH | 52 | HLA-DQB2 | 77 | PIKFYVE | 102 | TRAF6 |
| 3 | AP3B1 | 28 | CTSL | 53 | HLA-DRA | 78 | PSMB8 | 103 | TREM2 |
| 4 | AP3D1 | 29 | CTSS | 54 | HLA-DRB1 | 79 | PSME1 | 104 | TREX1 |
| 5 | ARL8B | 30 | CTSV | 55 | HLA-DRB3 | 80 | PYCARD | 105 | WAS |
| 6 | ATG5 | 31 | DNM2 | 56 | HLA-DRB4 | 81 | RAB10 | 106 | WDFY4 |
| 7 | AZGP1 | 32 | ERAP1 | 57 | HLA-DRB5 | 82 | RAB27A | 107 | YTHDF1 |
| 8 | B2M | 33 | ERAP2 | 58 | HLA-E | 83 | RAB32 |  |  |
| 9 | CALR | 34 | EXT1 | 59 | HLA-F | 84 | RAB33A |  |  |
| 10 | CCL19 | 35 | FCER1G | 60 | HLA-G | 85 | RAB34 |  |  |
| 11 | CCL21 | 36 | FCGR2B | 61 | HLA-H | 86 | RAB35 |  |  |
| 12 | CCR7 | 37 | FGL2 | 62 | ICAM1 | 87 | RAB3B |  |  |
| 13 | CD1A | 38 | GBA | 63 | IDE | 88 | RAB3C |  |  |
| 14 | CD1B | 39 | HFE | 64 | IFI30 | 89 | RAB4A |  |  |
| 15 | CD1C | 40 | HLA-A | 65 | IKBKB | 90 | RAB5B |  |  |
| 16 | CD1D | 41 | HLA-B | 66 | KDM5D | 91 | RAB6A |  |  |
| 17 | CD1E | 42 | HLA-C | 67 | LGMN | 92 | RAB8B |  |  |
| 18 | CD20 | 43 | HLA-DMA | 68 | LILRB2 | 93 | RELB |  |  |
| 19 | CD68 | 44 | HLA-DMB | 69 | LNPEP | 94 | RFTN1 |  |  |
| 20 | CD74 | 45 | HLA-DOA | 70 | MARCHF1 | 95 | SAR1B |  |  |
| 21 | CD8A | 46 | HLA-DOB | 71 | MARCHF8 | 96 | SLC11A1 |  |  |
| 22 | CLEC4A | 47 | HLA-DPA1 | 72 | MFSD6 | 97 | TAP1 |  |  |
| 23 | CLEC4M | 48 | HLA-DPB1 | 73 | MR1 | 98 | TAP2 |  |  |
| 24 | CTSD | 49 | HLA-DQA1 | 74 | NOD1 | 99 | TAPBP |  |  |
| 25 | CTSE | 50 | HLA-DQA2 | 75 | NOD2 | 100 | TAPBPL |  |  |

**Table S4** The details for 15 kinds of prediction models via machine learning integration (combined Lasso regression, StepCox, survivalSVM, RandomForest and Logistic) and further calculated the C-index of each model across all validation datasets (TCGA cohort, GSE14520 cohort, GSE76427 cohort and FAHWMU cohort).

| **Variables** | **TCGA (N = 365)** | **GSE14520 (N=221)** | **GSE76427 (N=115)** | **FAHWMU (N = 104)** | **Mean C-index** |  |
| --- | --- | --- | --- | --- | --- | --- |
|  |  |  |  |  |  |  |
| **Lasso** | 0.79 | 0.73 | 0.68 | 0.62 | 0.705 |  |
| **Lasso + StepCox** | 0.82 | 0.78 | 0.72 | 0.87 | 0.7975 |  |
| **Lasso + survivalSVM** | 0.7 | 0.72 | 0.81 | 0.69 | 0.73 |  |
| **Lasso + RF** | 0.83 | 0.75 | 0.65 | 0.66 | 0.7225 |  |
| **Lasso + Logistic** | 0.75 | 0.63 | 0.81 | 0.85 | 0.76 |  |
| **StepCox** | 0.65 | 0.61 | 0.71 | 0.75 | 0.68 |  |
| **StepCox + survivalSVM** | 0.62 | 0.65 | 0.68 | 0.82 | 0.6925 |  |
| **StepCox + RF** | 0.76 | 0.63 | 0.79 | 0.65 | 0.7075 |  |
| **StepCox + Logistic** | 0.72 | 0.64 | 0.69 | 0.67 | 0.68 |  |
| **survivalSVM** | 0.68 | 0.77 | 0.83 | 0.64 | 0.73 |  |
| **survivalSVM + RF** | 0.65 | 0.69 | 0.81 | 0.71 | 0.715 |  |
| **survivalSVM + Logistic** | 0.67 | 0.69 | 0.7 | 0.67 | 0.6825 |  |
| **RF** | 0.81 | 0.58 | 0.68 | 0.62 | 0.6725 |  |
| **Logistic** | 0.68 | 0.64 | 0.65 | 0.56 | 0.6325 |  |
| **RF + Logistic** | 0.86 | 0.69 | 0.75 | 0.67 | 0.7425 |  |
